# Pre-Silicon Accurate SPICE Modeling of Trench MOSFETs via Advanced TCAD Simulations and Dynamic Validation

**DOI:** 10.3390/mi16080955

**Published:** 2025-08-19

**Authors:** Ammar Tariq, Giovanni Minardi, Valeria Cinnera Martino, Enza Fazio, Salvatore Rinaudo, Giuseppe Privitera, Fortunato Neri, Carmelo Corsaro

**Affiliations:** 1 Department of Mathematical and Computer Sciences, Physical Sciences and Earth Sciences (MIFT), University of Messina, 98166 Messina, Italy; enza.fazio@unime.it (E.F.); fneri@unime.it (F.N.); 2 STMicroelectronics, 95125 Catania, Italy; giovanni.minardi@st.com (G.M.); valeria.cinnera@st.com (V.C.M.); salvatore.rinaudo@st.com (S.R.); giuseppe.privitera@st.com (G.P.)

**Keywords:** SPICE model, UTMOST IV, victory, process, device, PowerMOS

## Abstract

This work presents a novel and fully virtual flow for extracting the SPICE model of a power MOSFET, starting exclusively from TCAD simulations. Unlike traditional approaches that rely on experimental silicon data, our methodology enables designers to optimize the device performance and extract accurate electrical parameters before any physical prototyping is required. By leveraging advanced TCAD tools, we generate a realistic device structure and obtain all the key electrical characteristics, which are then used for precise SPICE model extraction and macromodel integration. The extracted model is dynamically validated using a gate-charge test performed identically in both the TCAD and SPICE environments, demonstrating excellent agreement with less than a 2% error in the charge quantities, Q_gs_ and Q_gd_. This approach proves that initial silicon prototyping can be confidently bypassed, and it is highly innovative because it enables designers to achieve highly faithful device simulations before hardware fabrication. This significantly reduces the need for costly and time-consuming prototyping and design re-spins, accelerating the development process while enhancing the accuracy in terms of the transient and dynamic characteristics of MOSFETs designed for specific applications; in our case, for an e-fuse to be integrated into a more complex system.

## 1. Introduction

Silicon metal–oxide–semiconductor field-effect transistors (MOSFETs) are fundamental in contemporary electronic systems owing to their scalability, versatility, and efficiency [1,2]. They play a crucial role in the development of various technologies, ranging from consumer electronics to advanced communication systems [3,4]. MOSFETs are widely used in applications such as switching, voltage regulation, and power management because of their ability to efficiently control electrical power [5,6]. Their importance is further underscored by their scalability to nanoscale dimensions, their role in high-frequency applications, and their potential in energy conversion and detection technologies [7]. However, as the device architecture evolves, particularly with the introduction of complex structures like trench-gate MOSFETs, fabrication complexity and reliability concerns such as self-heating and hot-carrier effects have become more pronounced [8,9]. These challenges necessitate ongoing research to optimize the device designs and ensure robust operation across diverse applications [10]. To accurately simulate and analyze the performance of these advanced MOSFETs, the extraction of SPICE models is crucial. SPICE models provide a detailed representation of the electrical characteristics of MOSFETs, which is essential for designing and optimizing electronic circuits [11,12]. Accurate SPICE models allow for precise performance evaluation, design optimization, and predictive capability under various conditions. Traditionally, SPICE parameter extraction relies on experimental measurements from fabricated silicon devices. Tools such as IC-CAP [13], BSIMPro [14], and Silvaco’s UTMOST are commonly used for SPICE parameter extraction. These simulators automate the extraction process by fitting simulation data to experimental measurements, ensuring that the models closely match the real-world performance and thus enhancing the reliability and efficiency of electronic circuit designs [15,16,17,18,19,20]. Nevertheless, this conventional workflow remains dependent on physical prototyping, which is time-consuming and costly, especially when multiple design iterations are required. Ultimately, improved SPICE modeling directly contributes to faster innovation cycles and more efficient power conversion designs, which is vital given the growing demand for energy-efficient electronics and the complexity of modern devices. SPICE modeling requires high computational resources and time for very complex circuits, limiting its scalability. Additionally, the accuracy of the models depends heavily on the quality and completeness of the input parameters and device characterization data. Therefore, while improved SPICE modeling accelerates innovation and design efficiency, it should be complemented by experimental validation and other modeling techniques to ensure robust results. To overcome these limitations, there is a growing shift toward virtual prototyping methodologies. Technology computer-aided design (TCAD) tools now enable highly accurate simulation of device physics, allowing for the extraction of electrical parameters and performance metrics without the need for initial silicon fabrication [21].

Integrating TCAD data directly into SPICE model extraction, and validating these models dynamically, represents a significant advancement in power device design. In this study, we present a novel and fully virtual workflow for extracting and validating SPICE models of trench-gate power MOSFETs, utilizing Silvaco’s UTMOST IV and advanced TCAD simulations. Unlike traditional approaches, our method bypasses the need for initial silicon prototyping by leveraging physically based TCAD data as the foundation for model extraction. The extracted models are dynamically validated through gate-charge (Q_g_) testing performed identically in both the TCAD and SPICE environments, ensuring robust prediction of real-world switching behavior. As demonstrated in this work, this approach enables reliable pre-silicon optimization, significantly reducing the development time and cost, and demonstrates that accurate, predictive circuit models for advanced power MOSFETs can be achieved entirely in silicon.

In this work, our study employs a conventional MOSFET as a test case to rigorously assess and validate a simulation methodology, utilizing advanced TCAD simulations combined with dynamic validation, specifically aimed at the development of an e-fuse, a critical protection device for high-value electronic components. The primary objective is to demonstrate the effectiveness and reliability of this methodology in a practical application, showcasing its potential to drive significant advancements and innovative breakthroughs in the field of microelectronics. Upon successful validation with the traditional MOSFET, this approach holds promise for extension to a wide range of other electronic devices, paving the way for future innovations and enhanced device design.

By leveraging advanced TCAD tools, we generate realistic device structures and extract key electrical characteristics, which serve as the foundation for precise SPICE model extraction and macromodel integration. The extracted models undergo dynamic validation via identical gate-charge tests performed in both the TCAD and SPICE environments, demonstrating excellent agreement with less than a 2% error in the charge quantities, Q_gs_ and Q_gd_ (our critical switching parameters). The advantage of using the gate-charge test for validation is that the designer can easily calculate the amount of current required from the drive circuit to switch the device “on” in the desired length of time (because Q = CV and I = C dV/dt, the gate charge is the product of the time and current); the time needed to turn a MOSFET “on” corresponds to the time required to inject the two data sheet charge quantities, Q_gs_ and Q_gd_, into the gate. So, by using this approach, the initial silicon prototyping can be confidently bypassed, yielding substantial reductions in the development time and cost, while ensuring robust pre-silicon optimization of power MOSFETs. The central innovation lies in seamlessly bridging physics-based TCAD simulations directly with SPICE compact model extraction, eliminating the need for costly and time-consuming silicon fabrication iterations. This virtual prototyping framework allows for precise parameter extraction informed by first-principles device physics, enabling predictive modeling of device behavior across diverse operating conditions prior to manufacture. The dynamic cross-validation technique enhances the predictive confidence of the extracted models, significantly improving the fidelity of circuit-level simulations and facilitating optimized design cycles with fewer costly experimental iterations. Thus, although the presented data are foundational rather than groundbreaking, this approach supports the development of devices with reduced switching losses, enhanced thermal stability, and greater operational reliability in demanding environments. Such progress is poised to benefit a broad range of applications, including power converters, electric vehicles, renewable energy systems, and high-frequency communication technologies.

## 2. TCAD Simulation

As is well known [22,23,24,25,26], TCAD simulations are essential for understanding the physical behavior of semiconductor devices. For a high-voltage power MOSFET, TCAD simulations help in obtaining accurate electrical characteristics. They enable optimized design through virtual testing of materials, architectures, and process variations that are costly or impractical to prototype. TCAD supports detailed 2D/3D modeling of complex structures, accounting for the dopant distribution, stress, and electrical noise, which affect device performance. This capability is essential for enhancing the power, performance, and reliability while reducing the development time and cost. Furthermore, TCAD facilitates scaling and innovation in novel device architectures by providing precise multi-dimensional simulations critical at a nanoscale, ensuring optimized high-voltage operation in power semiconductors.

**Figure 1:**
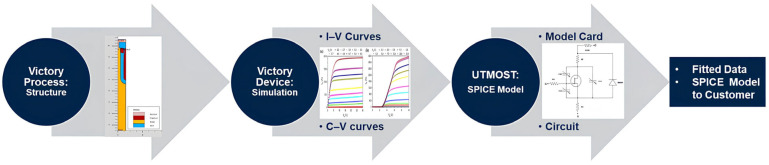
TCAD to SPICE flow of PowerMOS.

In our work, to predict these characteristics and optimize the device performance, a TCAD to SPICE flow was implemented (Figure 1). This flow ensures that the data obtained from the TCAD simulations is effectively used for the subsequent SPICE model extraction, which is crucial for the design and optimization phases of the device development process. In our proposed pre-silicon SPICE modeling workflow, TCAD is not used solely for qualitative analysis but also as a quantitatively accurate source of I–V and C–V data for BSIM3 parameter extraction, enabling model fitting and dynamic validation without the need for initial silicon prototyping.

### 2.1. Process Structure

**Figure 2:**
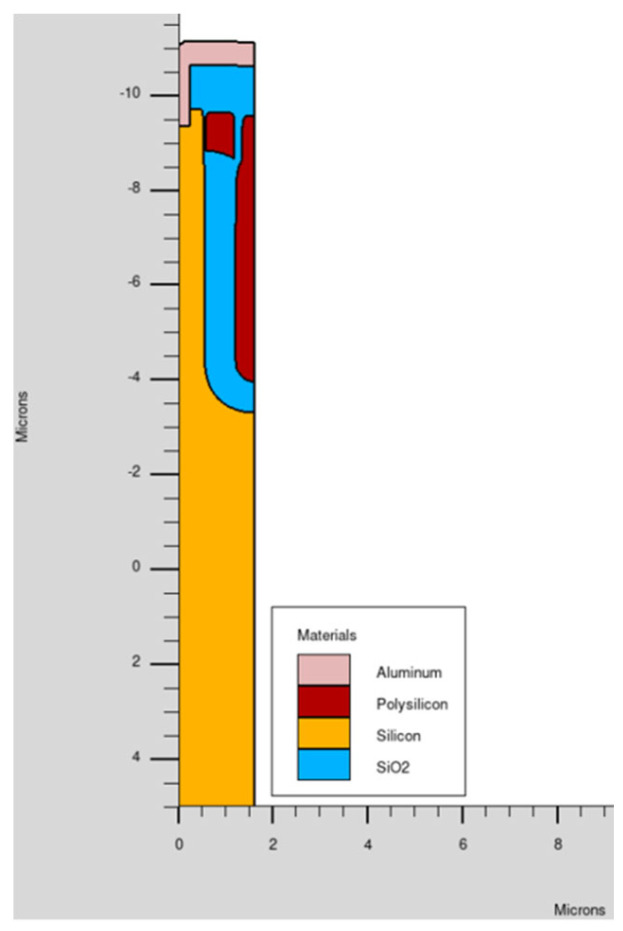
Process structure of trench MOSFET.

The process simulation for the trench-gate MOSFET begins with the precise definition of its two-dimensional geometry (Figure 2) using the Victory Process tool, based on the STMicroelectronics SPG100 (VP33) technology node under development and whose main application is an e-fuse (DC application). Critical structural parameters such as the trench depth and width, gate oxide thickness, channel length, and doping concentrations are carefully specified to replicate the actual fabrication process. The simulation workflow incorporates essential process steps, including oxidation, trench etching, polysilicon gate deposition, ion implantation, and thermal annealing. These steps determine the final doping profiles, junction depths, and physical dimensions of the device, all of which have a direct impact on the MOSFET’s electrical characteristics, including the threshold voltage, on-resistance, and breakdown voltage. By accurately modeling these parameters, the TCAD process simulation generates a realistic device structure that serves as the foundation for subsequent device-level simulations. This approach ensures that the extracted electrical data used for the SPICE modeling is both physically meaningful and predictive, enabling robust device optimization and reliable pre-silicon validation before any physical prototyping is undertaken.

### 2.2. Device Simulations

By utilizing the Victory Device tool, starting from the full physical description of our MOSFET (Figure 2), we performed detailed DC and AC simulations to obtain comprehensive I–V and C–V characteristics pertaining to the power MOSFET across varying conditions. For instance, I_D_-V_D_: (V_D_ up to 30 V) @ V_G_ ranging from 2.5 to 5.5 V (11 steps), I_D_-V_G_: (V_G_ up to 10 V) @ V_D_ = 0.1, 0.3, 0.5, 1, 2, 3, 5, 7, 10, 20, 30, reverse characteristics of the body-drain diode @ V_G_ = −2 V and capacitance curves were obtained. In terms of the interterminal capacitances, in the model we used C_iss_, C_oss_ and C_rss_.

**Figure 3:**
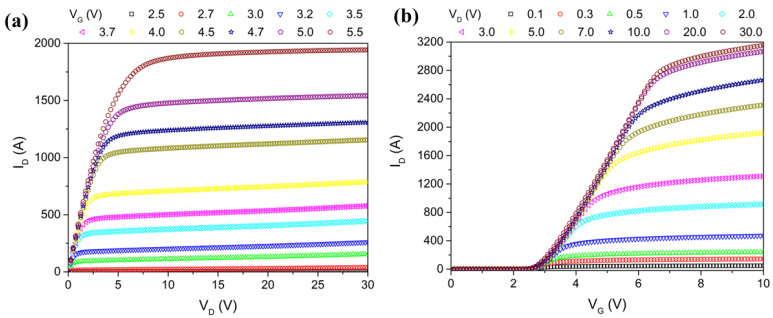
(**a**,**b**) I–V characteristics and transfer characteristic of the trench MOSFET (I_D_ and V_D_ values for V_g_ ranging from 2.5 V to 5.5 V. The I–V characteristic range is purely theoretical and used only for model extraction; in practice, the MOSFET device, due to its physical size, cannot sustain currents above 60 A.

DC simulations are essential for obtaining the current–voltage characteristics associated with a power MOSFET. These I–V characteristics provide insights into the current–voltage behavior, which is essential for understanding the device’s switching and conduction properties at varying operating conditions up to the breakdown of the device (Figure 3a,b). Ultimately, if well known, I–V characteristics serve as the quantitative input datasets for BSIM3 parameter extraction in our TCAD-to-SPICE modeling flow. We have also explained that the wide V_GS_ and V_DS_ sweep ranges ensure coverage of both low-field and high-field effects, enabling accurate model fitting across the entire operating range of the trench-gate MOSFET. We remark that our methodology aligns well with advanced TCAD device characterization practices commonly employed in the field, especially in works using tools like Victory Device [27,28]. The I–V and C–V profiles (well established for trench-gate MOSFETs) are appropriately generated, parameterized, and dynamically validated entirely through a pre-silicon TCAD-to-SPICE extraction flow, without any experimental measurements. Thus, the novelty lies in the methodology adopted consisting of a fully virtual framework that accurately produces and validates SPICE-compatible models before fabrication. This eliminates the dependency on initial silicon prototypes, reducing the development cost and time while preserving the modeling fidelity.

**Figure 4:**
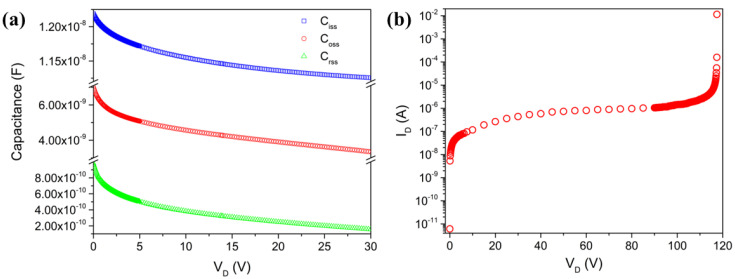
C–V characteristics: C_iss_, C_rss_, and C_oss_, (**a**) and breakdown voltage (**b**).

Furthermore, AC simulations are performed to obtain the capacitance–voltage (C–V) characteristics of the power MOSFET. These characteristics are important for understanding the parasitic capacitances that affect the switching behavior and frequency response of the device (Figure 4a). The input capacitance (C_iss_ = C_gs_ + C_gd_), output capacitance (C_oss_ = C_ds_ + C_gd_), and reverse transfer capacitance (C_rss_ = C_gd_) all exhibit a typical decreasing trend with increasing drain voltage (V_D_). This behavior is due to the expansion of the depletion region at higher V_D_, which causes the capacitance to decrease. Specifically, the initial values of C_iss_, C_oss_, and C_rss_ are approximately 10.8 nF, 6 nF, and 4.4 nF at 0 V, decreasing to approximately 9.6 nF, 3.5 nF, and 3.2 nF at 24 V, respectively. These decreasing trends indicate that higher drain voltages require less charge to switch the MOSFET, which can affect the switching speed and high-frequency performance. Additionally, the breakdown voltage (BV) of the MOSFET is a critical parameter that defines the maximum voltage the device can withstand before it undergoes avalanche breakdown. When the V_D_ exceeds the BV, a significant increase in the drain current (I_D_) is observed, indicating the onset of breakdown. This behavior is evident in the provided graph (Figure 4b), where the I_D_ sharply increases at the BV point. Ensuring that the MOSFET operates below its BV (in our case, a MOSFET with a BV greater than 100 V) is crucial for the reliability and longevity of the device, especially in high-voltage applications where a higher BV is essential to prevent device failure and to optimize the switching performance and efficiency.

## 3. Results

### 3.1. Initial Parameter Extraction

In this step, the data obtained from the TCAD simulations are used to extract the initial parameters for the BSIM3 model [21]. The extracted data will serve as input to the UTMOST IV model extraction tool, which is specifically designed for parameter extraction and model fitting. The key parameters to extract are as follows.

**Figure 5:**
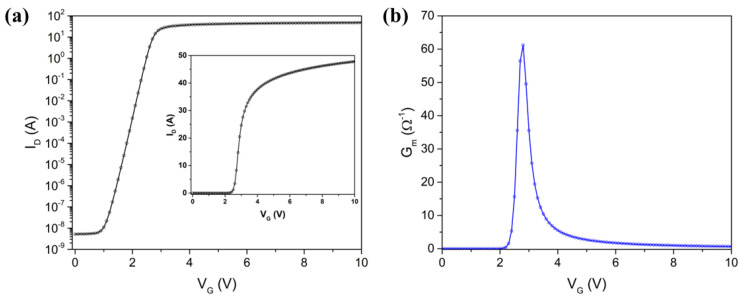
(**a**) Threshold, transfer characteristic (the inset reports a linear scale) and (**b**) G_m_ (V_DS_ = 0.1 V).

**Threshold voltage (Vth):** The gate voltage (V_G_) at which the MOSFET begins to conduct a significant amount of current. To extract the Vth, we analyze the transfer characteristics, which represent a graphical depiction of the I_D_ in relation to the gate-source voltage (V_GS_) (Figure 5a). During this analysis, we look for the point on the curve where the I_D_ starts to increase rapidly. This point indicates the V_G_ where the MOSFET shows transitions from the off state to the on state, marking Vth. In this case, Vth is approximately 3 V.

**Mobility (µ):** It is the movement capability of charge carriers through the semiconductor material. To calculate the mobility, we focus on the linear region of the transfer characteristics (Figure 5a). In this region, the MOSFET operates in the linear (ohmic) mode. By analyzing the slope of the I_D_ vs. V_GS_ curve, we determine the mobility. The transconductance (G_m_) is a measure of the change in the I_D_ with respect to the change in V_GS_ (1), as given by:

(1) $$G_{m}=\frac{\partial I_{D}}{\partial V_{GS}}$$

The maximum G_m_ is typically observed in the linear region and is used to calculate the µ. The steeper the slope, the higher the mobility, indicating that charge carriers can move more easily through the semiconductor material. From Figure 5b, the peak value of G_m_ is approximately 60 mS. Using the peak G_m_ value and typical values for the device parameters, the estimated mobility is approximately 0.02147 m^2^/Vs.

**Channel length modulation (λ):** The variation of the effective channel length with the drain voltage (V_D_), which affects the output conductance. We analyze the output characteristics between the I_D_ and the drain-source voltage (V_DS_) at varying V_GS_. We observe how the I_D_ responds as the V_DS_ increases, while keeping the V_GS_ constant. In the saturation region, ideally, the I_D_ should remain constant as the V_DS_ increases. However, due to λ and the drain-induced barrier-lowering (DIBL) effect, the effective channel length and Vth decrease, causing a slight rise in the I_D_ with increasing V_DS_. By examining the slope of the I_D_ vs. V_DS_ curves at various gate voltages, we can quantify these effects. From Figure 3a, we identify the saturation region at higher V_DS_ values (e.g., V_DS_ > 10 V). We select two points in the saturation region to calculate the slope: I_D_ ≈ 2000 µA at V_DS_ ≈ 15 V and I_D_ ≈ 2100 µA at V_DS_ ≈ 20 V. The slope calculation is ΔI_D_/ΔV_DS_ = 20 µA/V. Extrapolating this slope to intercept the *x*-axis, we find the early voltage (VA) to be approximately 50 V. Using the formula = 1/VA, we calculate the channel length modulation (λ) to be 0.02 V^−1^.

The **subthreshold slope (S)** is the rate at which the I_D_ rises with the V_G_ in the subthreshold region of the transfer characteristics. We emphasize that, in this work, the primary focus is on extracting and modeling the subthreshold slope (SS) from TCAD-simulated device characteristics, rather than optimizing the SS through design modifications. This approach is justified because the target e-fuse application primarily operates in DC conditions, where the switching speed is not critical. The key parameter is the turn-on behavior, which should be sufficiently gradual to prevent oscillatory ringing. Consequently, the circuit can tolerate relatively high capacitances and an extended Miller plateau duration. In the subthreshold region, the MOSFET remains in a partially conductive state, leading to an exponential increase in current with the V_G_. We plot the log (I_D_) against the V_GS_. The subthreshold slope is then determined by calculating the slope of the log (I_D_) vs. V_GS_ plot in the subthreshold region. In the context of the BSIM3 model, the subthreshold behavior is described by the following parameters. NFACTOR: this parameter represents the subthreshold swing factor, which affects the steepness of the subthreshold slope. VOFF: this parameter represents the offset voltage, which adjusts the Vth in the subthreshold region. The initial fitting process involves using the extracted data to perform a preliminary adjustment of the main model parameters. This step provides an estimate of the key parameters, such as the Vth, μ, λ, and S. By inputting these initial values into the BSIM3 model, we can begin to approximate the behavior of the MOSFET. The initial fitting provides a foundation, but to ensure the model accurately represents the real device behavior, we must iteratively refine and optimize a wide range of BSIM3 parameters.

#### Macromodel Integration

**Figure 6:**
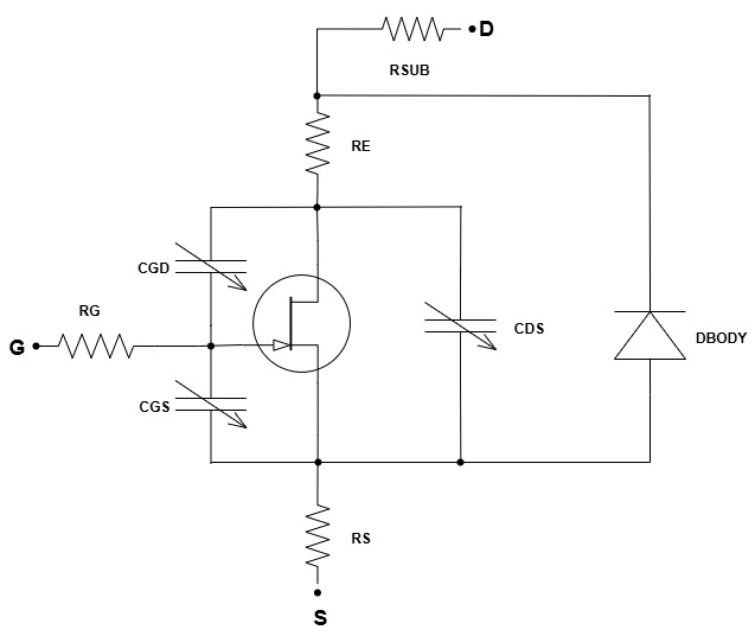
Macromodel used for model extraction.

The macromodel (Figure 6) provides a higher-level abstraction that incorporates additional elements to simulate the complex behaviors and parasitic effects inherent in the power MOSFET used as the test case (Figure 2), which are not fully captured by the standard BSIM3 model.

Components of the macromodel:

1. Gate network:
  - R_G_: resistor representing the gate parasitic resistance.
  - C_GD_, C_GS_, C_DS_: capacitors representing the parasitic capacitances between the gate-drain and the gate-source and drain-source.
2. Transistor core:
  - NMOS: the main MOSFET device modeled using BSIM3 parameters.
3. Source and drain networks:
  - R_E_: external source resistance.
  - R_SUB_: parasitic resistances in the drain and substrate.
  - DBODY: body diode and its associated resistance.

The parasitic inductances in series with the resistances (generally considered as reported in Ref. [29]) are initially neglected in the circuit model to simplify the analysis and focus on the dominant device capacitances and resistances. These inductances become significant primarily under high-frequency or fast-switching conditions, where they can cause voltage overshoots and additional losses. Since the application we are evaluating (an e-fuse under DC conditions) does not involve fast switching, the parasitic inductances are not taken into account.

**Figure 7:**
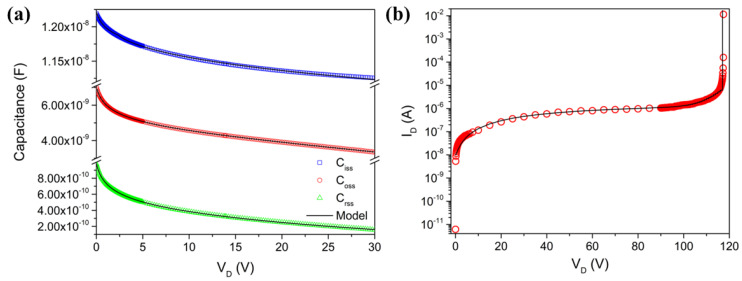
(**a**) Capacitance curves, respectively, Ciss, Coss and Crss. (**b**) Diode body-drain reverse mode.

Below, we outline the steps taken to implement the procedure. The initial step in incorporating the macromodel involves the identification and definition of the sub-models required to represent the various aspects of the device. These sub-models encompass elements such as the parasitic resistance and capacitance. For instance, we need to account for the gate resistance (R_G_), the parasitic resistance in the source and drain (R_SG_, R_SD_) and the parasitic capacitances between the gate and drain (C_GD_) and between the gate and source (C_GS_). By defining these sub-models, we can capture the detailed electrical characteristics that influence the device’s performance. With the macromodel defined, we proceed to extract parameters for each sub-model using the data from the TCAD simulations and the initial fitting results. This step involves detailed analysis and fitting to ensure that each sub-model accurately represents the corresponding aspect of the device. To further illustrate the importance of these sub-models, we can include all the fitted plots that highlight key characteristics of the MOSFET (Figure 7). In Figure 7b, the reverse characteristics of the body-drain diode, including the breakdown voltage, are fitted. Similarly, Figure 7a shows the fitted (black lines) and simulated (symbols) capacitance curves. It is essential for understanding the switching characteristics and dynamic performance of the device. The result shows excellent fitting, with an error less than 1%.

**Figure 8:**
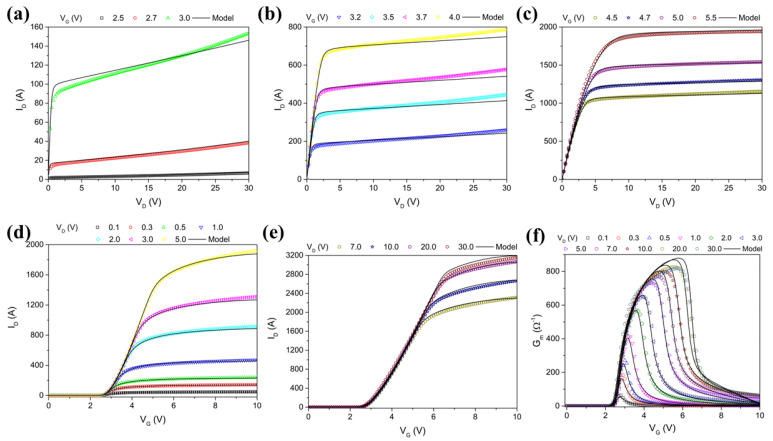
Fitted IDVD (**a**–**c**), IDVG (**d**,**e**) and Gm (**f**) curves by UTMOST IV.

**Figure 9:**
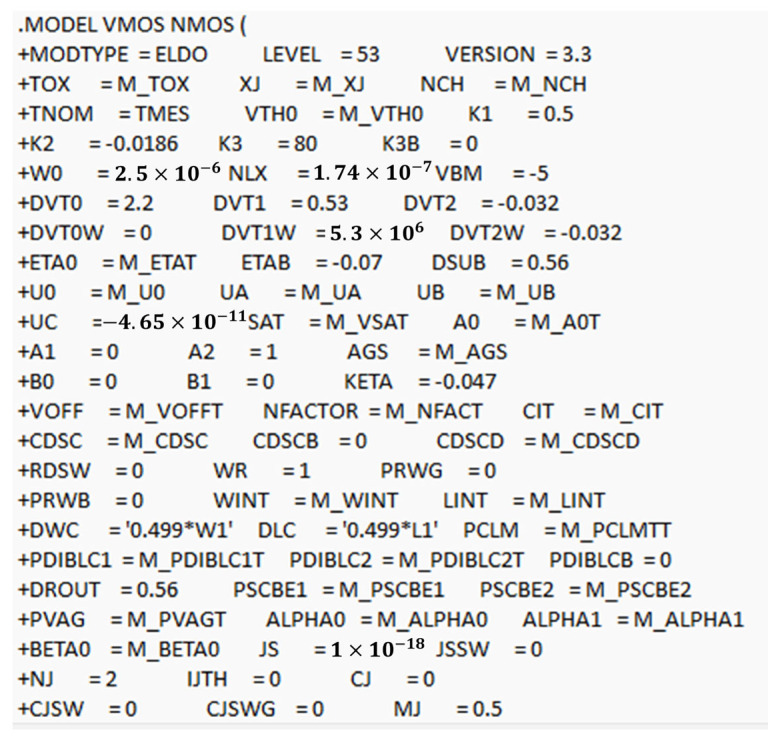
UTMOST IV partial list of extracted BSIM3 SPICE parameters. The PSpice Model Editor supports editing BSIM3 model parameters through direct text edits or importing model files.

The final step is to validate the combined macromodel against the reference TCAD simulation data. This involves comparing the simulated results from the macromodel with the characteristics of the device obtained by TCAD simulation. By doing so, we can ensure that the macromodel accurately represents the device behavior under various operating conditions. Figure 8 shows the comparison of I_D_ vs. V_D_ for the simulation (symbols) and fitted model (lines) at low, medium, and high V_DS_ values, as well as the comparison of the I_D_ vs. V_G_ and *G_m_* curves. We can see from this result that excellent fitting was achieved, with minimum errors for all the curves. These comparisons are crucial for validating the macromodel’s accuracy. Validation is an iterative process, where we refine the parameters and sub-models as needed to achieve a close match between the simulated and measured data. This step is essential for confirming the reliability and accuracy of the micromodel. Once the macromodel has been validated, the next step is to generate the SPICE file that includes all the components and parameters of the macromodel. This file serves as a comprehensive representation of the power MOSFET, incorporating both the core BSIM3 model and the additional elements that account for parasitic effects and other complex behaviors. The process involves translating the validated macromodel into a SPICE-compatible format (Figure 9). This includes defining the subcircuits and specifying the values for all the components based on the extracted and refined parameters. The final step is to integrate the SPICE file of the macromodel into the simulation environment. This involves importing the SPICE file into tools such as HSPICE and LTspice. Specifically, we employ the ELDO simulator, integrating it within the framework of a full circuit design.

Summarizing, the process begins by loading the SPICE file containing the macromodel into the simulator, enabling the tool to recognize and utilize the model’s defined components and parameters during simulation. After importing the macromodel, it is integrated into the complete circuit schematic by establishing appropriate connections with other circuit elements. Once integrated, the macromodel functions within the full circuit context during simulation, exhibiting the behavioral characteristics specified in the SPICE file. Subsequently, the simulation settings are configured to ensure the environment executes the simulation using the defined model and circuit parameters, allowing for accurate analysis of the output results. Overall, this workflow typically involves creating or opening a schematic, importing the SPICE netlist or model file, assigning the model to relevant components if required, and setting up the simulation profile with the desired analysis parameters. Following verification, running the simulation generates output data that reflects the behavior of the complete circuit, including the macromodel. The PSpice Model Editor, which generates the model definitions and parts directly used in the PSpice schematic simulations for analyzing circuit behavior, is illustrated in Figure 9, showing the main schematic as an example.

### 3.2. Gate-Charge Test Simulation and Model Validation

**Figure 10:**
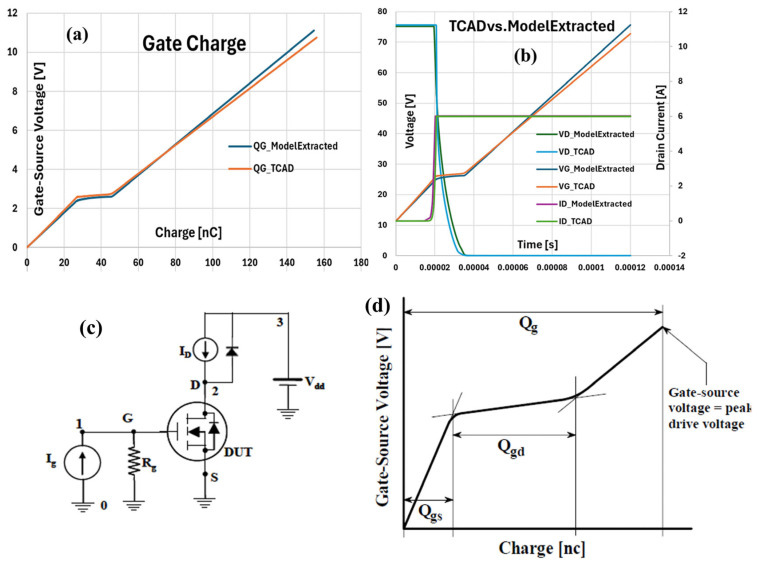
(**a**) Gate-to-source voltage versus total gate charge curve and (**b**) time-domain switching waveforms of V_G_, V_D_, and I_D_ during the gate-charge test for both the TCAD simulation and the extracted SPICE model. (**c**) Test circuit for Qg simulation, in which numbers identify relevant nodes. (**d**) Parts of the gate charge: *Qg*, the total gate charge, Q_gs_, the gate source charge, and Q_gd_, the gate-drain (“Miller”) charge.

To ensure the accuracy and reliability of the extracted SPICE model for the understudy trench-gate MOSFET, a dynamic validation was conducted by performing a gate-charge (Q_g_) test using both the TCAD mixed-mode simulation and the SPICE model. The gate-charge test is a standard technique to evaluate the total charge required to fully switch on the device, which directly impacts the switching speed, gate driver design, and overall efficiency in power electronic applications. The advantage of using the gate charge is that the designer can easily calculate the amount of current required from the drive circuit to switch the device on in the desired length of time. The physical structure of the MOSFET was first generated using Silvaco Victory Process, accurately replicating the real-world fabrication steps. This structure was then imported into Victory Device for electrical simulation. A mixed-mode simulation was implemented, where the device under test (DUT) was embedded in a standard gate-charge test circuit (Figure 10c). The gate was driven by a constant current source (I_G_ = 1.3 mA), the drain was biased at V_DD_ = 75 V, and the drain current was set to I_D_ = 6 A. The same test conditions were applied to both the TCAD device and the extracted SPICE model to ensure a fair and direct comparison.

Figure 10a shows the gate-source voltage (V_GS_) as a function of the total gate charge (Q_g_) for both the TCAD simulation and the SPICE model performed with the test circuit reported in Figure 10c. The curve can be divided into three distinct regions: the initial linear segment (Q_gs_), the Miller plateau (Q_gd_), and the final rise to the maximum gate voltage, which together represent the total gate charge required to fully enhance the device (Figure 10d).

In the Q_gs_ region, V_GS_ increases linearly with Q_g_, reflecting the charging of the gate-source capacitance. The subsequent Miller plateau is characterized by a nearly constant V_GS_ while the Q_g_ continues to increase, corresponding to the period when the gate current is primarily responsible for sweeping the drain voltage. This region is crucial for switching performance, as it dominates the switching loss and speed. The total Q_g_, observed at V_GS_ ≈ 11 V, represents the total charge needed for full device turn-on. The mathematical relationship governing the gate-charge test is: Q = C × V, where Q is the gate charge, C is the gate capacitance, and V is the gate-source voltage. During the Miller plateau, the plateau length directly relates to the device’s gate-drain (Miller) capacitance, which is a key parameter for dynamic switching analysis. The key parameters were extracted from the gate-charge curves for both the TCAD and the SPICE model. The calculated Q_gs_ was found to be 27 nC in the TCAD and 28 nC in the SPICE model. The Q_gd_ values were 40 nC and 39 nC, respectively. Finally, the total gate charge (Q_g_) required for full device turn-on was 156 nC for the TCAD and 154 nC for the SPICE model. These minor differences, within approximately 1–2%, confirm the high accuracy of the extracted SPICE model in replicating the device’s dynamic switching behavior. Similarly, Figure 10b presents the time-domain switching waveforms for both the TCAD simulation and the extracted SPICE model, including the V_G_, V_D_, and I_D_. As the gate current charges the device, the V_G_ rises smoothly and almost identically in both approaches, demonstrating that the gate capacitance and charging dynamics are accurately captured by the model. Once the V_G_ reaches the threshold, the MOSFET turns on, resulting in a sharp drop in the drain voltage from V_DD_ toward a lower value; this transition occurs at nearly the same instant and with a similar slope in both the TCAD and SPICE results. Simultaneously, the drain current rises rapidly to the set value of 6 A, with both simulations showing an almost perfect overlap throughout the switching event. This close agreement across all three waveforms confirms that the extracted SPICE model reliably reproduces the dynamic switching behavior of the device as predicted by the advanced TCAD simulations.

## 4. Conclusions

In this study, we present an innovative and highly accurate SPICE modeling methodology for trench-gate MOSFETs, achieved by extracting the device parameters directly from the TCAD-simulated I–V and C–V characteristics using Silvaco’s UTMOST IV tool. By fitting the model parameters precisely to the TCAD-generated static (both DC and AC) data, the resulting SPICE model exhibits exceptional fidelity in replicating the device’s electrical behavior. Crucially, to rigorously validate the model’s predictive capability beyond mere curve fitting, we performed dynamic gate-charge measurements under identical switching conditions in both the TCAD simulations and the SPICE model, achieving remarkable agreement, with deviations below 2%, on critical performance metrics. This confirms the model’s robustness across both static and transient operational regimes. Our innovative TCAD-to-SPICE parameter extraction and validation workflow enables robust pre-silicon device optimization coupled with accurate circuit-level simulation, substantially accelerating the design cycle while reducing the development costs. By encapsulating detailed physical insights from TCAD into compact, circuit-ready models, this approach fosters the advanced design of next-generation devices and facilitates exploration of novel materials, thus representing a significant leap forward in device and circuit co-design.

Looking ahead, we plan to extend the model to incorporate temperature-dependent effects, device aging, high-frequency behavior, and parasitic elements, alongside comprehensive validation on emerging wide-bandgap semiconductor technologies such as SiC and GaN. Furthermore, integrating the statistical variability and modeling of complex switching scenarios will enhance the model’s applicability and predictive accuracy in real-world applications. Finally, the future integration of machine learning techniques promises to automate and refine the parameter extraction, elevate the device behavior prediction, and accelerate the simulation runtimes. AI-driven adaptive learning frameworks have the potential to optimize the model fidelity continuously and expand the frontier of device and circuit design exploration, marking a pioneering step toward the seamless co-optimization of device physics and circuit performance.

## Data Availability

Data is contained within the article.

## Abbreviations

The following abbreviations are used in this manuscript:

MOSFET	Silicon metal–oxide–semiconductor field-effect transistors
TCAD	Technology computer-aided design
DIBL	Drain-induced barrier-lowering
